# Antibody Recognition of Human Epidermal Growth Factor Receptor-2 (HER2) Juxtamembrane Domain Enhances Anti-Tumor Response of Chimeric Antigen Receptor (CAR)-T Cells

**DOI:** 10.3390/antib13020045

**Published:** 2024-06-07

**Authors:** Guangyu Zhou, Shengyu Fu, Yunsen Zhang, Shuang Li, Ziang Guo, Defang Ouyang, Tianlei Ying, Yinying Lu, Qi Zhao

**Affiliations:** 1 Institute of Translational Medicine, Cancer Centre, Faculty of Health Sciences, University of Macau, Taipa 999078, Macau SAR, China; yb77637@um.edu.mo (G.Z.); yb97640@connect.um.edu.mo (S.F.); yc07621@connect.um.edu.mo (Z.G.); 2 MoE Frontiers Science Center for Precision Oncology, University of Macau, Taipa 999078, Macau SAR, China; 3 Institute of Chinese Medical Sciences, University of Macau, Taipa 999078, Macau SAR, China; yunsenzhang666@163.com (Y.Z.); defangouyang@um.edu.mo (D.O.); 4 The Fifth Medical Center of the PLA General Hospital, Beijing 100036, China; lishuang941002@hotmail.com; 5 MOE/NHC/CAMS Key Laboratory of Medical Molecular Virology, Shanghai Institute of Infectious Disease and Biosecurity, Shanghai Engineering Research Center for Synthetic Immunology, School of Basic Medical Sciences, Fudan University, Shanghai 200032, China; tlying@fudan.edu.cn

**Keywords:** HER2, monoclonal antibody, CAR-T cell, trastuzumab

## Abstract

Chimeric antigen receptor (CAR) T cell therapy shows promise in treating malignant tumors. However, the use of human epidermal growth factor receptor-2 (HER2) CAR-T cells carries the risk of severe toxicity, including cytokine release syndrome, due to their “on-target off-tumor” recognition of HER2. Enhancing the quality and functionality of HER2 CARs could greatly improve the therapeutic potential of CAR-T cells. In this study, we developed a novel anti-HER2 monoclonal antibody, Ab8, which targets domain III of HER2, distinct from the domain IV recognition of trastuzumab. Although two anti-HER2 mAbs induced similar levels of antibody-dependent cellular cytotoxicity, trastuzumab-based CAR-T cells exhibited potent antitumor activity against HER2-positive cancer cells. In conclusion, our findings provide scientific evidence that antibody recognition of the membrane-proximal domain promotes the anti-tumor response of HER2-specific CAR-T cells.

## 1. Introduction

Breast cancer poses a significant health threat to women worldwide, with a distressingly high mortality rate. A pivotal player in breast cancer prognosis is the human epidermal growth factor receptor-2 (HER2), also known as Erbb2. As a member of the epidermal growth factor receptor family, HER2 boasts tyrosine kinase activity [1]. When ligands bind to HER2, they prompt the formation of homo- or heterodimer complexes, initiating tyrosine kinase activity. This activation, in turn, triggers cascades of downstream signaling pathways, including the MAP kinase, PI3K/AKT, and JAK/STAT pathways [2]. HER2’s role is indispensable in the development and differentiation of normal cells in various tissues, including epithelial, mesenchymal, and neuronal tissues. However, the aberrant expression or amplification of HER2 is observed in approximately 20–25% of breast cancers, profoundly implicated in dysregulated proliferation, invasion, and survival of cancer cells [3].

Monoclonal antibodies (mAbs) have become a cornerstone of breast cancer treatment in recent decades [4]. Trastuzumab, a humanized mAb, is widely used to treat HER2-positive breast cancer patients who do not respond to chemotherapy and endocrine therapy [5]. Trastuzumab targets the extracellular domain (ECD) IV of HER2, suppressing intracellular HER2 signaling pathways by inducing internalization and degradation of the HER2 receptor. Evidence confirms that trastuzumab mediates antibody-dependent cellular cytotoxicity (ADCC) against HER2+ tumor cells by recruiting cytotoxic, innate immune cells. However, resistance to trastuzumab can develop over time in a significant number of patients, reducing its effectiveness and necessitating the development of novel treatment strategies to improve survival rates among HER2+ breast cancer patients [6]. Pertuzumab is a humanized mAb that binds to the extracellular domain II of HER2. It prevents HER2 heterodimerization with HER1, HER3, and HER4, suppressing downstream PI3K, and MAPK pathways. Unlike trastuzumab, which binds to HER2 domain IV, pertuzumab mediates the effective ADCC effects by binding to HER2 domain II. Pertuzumab has been approved for use in combination with trastuzumab and docetaxel for first-line therapy in HER2+ breast cancer patients.

Chimeric antigen receptor (CAR)-T cell therapy has emerged as a promising approach for treating leukemia and certain solid tumors [7]. Typically, a CAR construct comprises an antibody variable region fragment (scFv), a transmembrane region, and intracellular signaling domains that activate T cells. CARs enable T cell activation independently of major histocompatibility complex (MHC) molecules. Donor-derived T cells are engineered to express multivalent CARs on their surface, which recognize tumor-associated antigens (TAAs) on tumor cells. The first anti-CD19 CAR T therapy has been approved for treating advanced leukemia in both children and adults. Clinical and preclinical trials of HER2-CAR T cells have been conducted in various HER2+ tumors, including breast cancer, ovarian cancer, glioblastoma, and colorectal cancer [8,9,10,11,12]. The clinical trials have validated the safety and efficacy of HER2-CAR-T cells in breast cancer therapy. Recent studies suggest that combining HER2-CAR-T cells may overcome resistance to trastuzumab in breast cancer cells [13,14]. However, the treatment of cancer patients with HER2-CAR-T cells can lead to serious adverse events due to inappropriate antigen recognition [15,16]. Therefore, the selection of appropriate HER2-specific CARs remains crucial for safe and effective therapy.

Interleukin (IL)-2 is a cytokine commonly used to expand therapeutic T cell products administered to patients [17]. However, the repeated stimulation of T cells with IL-2 during ex vivo expansion can lead to T cell exhaustion and decreased T cell persistence. IL-15, on the other hand, is produced as a stable heterodimer consisting of the IL-15 polypeptide single-chain bound to co-expressed IL-15 receptor α (IL-15Rα) [18]. Several IL-15 superagonists have been developed and evaluated in clinical trials. Although IL-2 and IL-15 share the same heterodimeric transducing receptor, IL-15 does not activate T regulatory cells or induce activation-induced cell death (AICD) in T cells. Some studies have demonstrated that IL-15 enhances the activities of anti-CD19 CAR-T cells by preserving their stem cell memory phenotype [19].

In this study, we generated a fully human mAb, Ab8, targeting HER2 from a human phage antibody display library. Ab8 recognizes a distinct epitope on HER2 compared to trastuzumab. Furthermore, we evaluated the antitumor effects of HER2-CAR-T cells derived from Ab8 and trastuzumab scFvs alone or in combination with IL-15 cytokine.

## 2. Materials and Methods

### 2.1. Cell Lines and Cell Culture

The SK-BR-3, MCF-7, MDA-MB-231, and BT474 cell lines were sourced from the Stem Cell Bank, Chinese Academy of Sciences (Shanghai, China). The HCC1954 cell line was obtained from MSKCC (New York City, NY, USA). The CTLL-2 cell line was obtained from RIKEN (Tokyo, Japan). These cell lines were cultured in RPMI-1640, DMEM, or DMEM/F12, supplemented with 10% fetal bovine serum (FBS) (GIBCO) (Grand Island, CA, USA), 100 U/mL penicillin, and 100 µg/mL streptomycin (GIBCO) at 37 °C with 5% CO_2_. The 293 freestyle cells were acquired from Invitrogen (Carlsbad, CA, USA) and were cultured in Freestyle 293 expression medium (GIBCO), within an incubator shaker set at 125 rpm, 8% CO_2_, and 37 °C.

### 2.2. Panning of a Human Antibody Phage Displaying Library

Panning of a human antibody phage display library was performed using recombinant human HER2 ECD protein (10004-H08H, SinoBiological, Beijing, China) as the target antigen as previously described with some modifications [20]. In brief, the human HER2 ECD protein was biotinylated using the EZ-Link Sulfo-NHS-Biotinylation Kit (Thermo Scientific, Waltham, MA, USA) according to the manufacturer’s instructions. The biotinylated protein was then conjugated onto Dynabeads M-280 (Invitrogen) to serve as the target for library panning. Initially, 10 µg of biotinylated antigen was used in the first round of panning, with 10^12^ amplified phages employed for the process. Following ten washes, bound phages on the beads were directly utilized to infect exponentially growing TG1 cells and rescued by the M13KO7 helper phage (NEB Biolabs, Ipswich, MA, USA). Panning was repeated for two additional rounds using 2 µg of biotinylated protein in each round. After the third round of panning, two hundred individual colonies were picked and identified. The plasmids from antigen-binding clones were subsequently isolated and sequenced to identify specific antibody sequences.

### 2.3. Antibody Production

The scFv fused with His and Flag tags at the C terminal were expressed and purified following previously established methods [21]. In brief, HB2151 cells were transformed with a pComb3x plasmid containing scFv sequences and expressed in the presence of 0.5 mM isopropyl-L-thio-β-D-galactopyranoside (Sigma-Aldrich, St Louis, MO, USA). The scFvs were purified using a Ni-NTA column (QIAGEN, Dusseldorf, Germany). For IgG1 expression, 293 FreeStyle cells were utilized as previously described [22]. The transfection of the cells was achieved using polyethylenimine (PEI; MW 25000, Polysciences, Warrington, PA, USA). IgGs were purified using Protein A resins (GE Healthcare, Chicago, IL, USA).

### 2.4. CTLL-2 Cell Proliferation Assay

To evaluate the activity of IL-15/Rα-Fc, CTLL-2 cells were seeded at a density of 50,000 cells per well in a 96-well plate. The IL-15/Rα-Fc complex was initially diluted to a concentration of 2 ng/mL, followed by serial ten-fold dilutions, and added to the wells in triplicate. Recombinant human IL-2 (Sihuan Pharm, Beijing, China) served as the positive control. The plate was then incubated at 37 °C with 5% CO_2_. After the designated incubation period, the plate was washed with PBS, and 10 μL of the Cell Counting Kit-8 (CCK-8) solution (Dojindo, Tabaru, Japan) was added to each well. Subsequently, the absorbance at 450 nm was measured using a plate reader (Molecular Device, Sunnyvale, CA, USA).

### 2.5. Enzyme-Linked Immunosorbent Assay (ELISA)

To screen phage clones binding to the HER2 antigen, a phage-ELISA was conducted using a 96-well ELISA plate from Corning. The plate wells were coated overnight at 4 °C with 50 ng of HER2 ECD in coating buffer. To block nonspecific binding sites, the wells were then incubated with PBS buffer containing 0.2% BSA at 37 °C for 1 h prior to the addition of phages. Simultaneously, individual phage colonies were randomly selected and inoculated into 2 × YT medium supplemented with 2% glucose and 100 µg/mL of ampicillin in a 96-well cluster plate from Corning. The plates were incubated at 37 °C for 6 h, followed by the addition of M13KO7 helper phage to the grown clones. After the 96-well cluster plates were incubated for 1 h, the infected bacterial cells were collected by centrifugation. The bacterial pellets were then resuspended in fresh 2 × YT medium containing 100 µg/mL ampicillin and 50 µg/mL of kanamycin. The bacterial culture was incubated overnight at 30 °C, and the culture medium containing the phage was harvested by centrifugation. Subsequently, 100 µL of the phage culture was added to each well in the ELISA plate and detected using HRP-conjugated mouse anti-M13 antibody. The reaction was initiated with the addition of the substrate solution of 3,3′,5,5′-Tetramethylbenzidine substrates (TMB) from Beyotime, and the absorbance was measured at 450 nm.

To evaluate antigen binding, 50 ng of HER2 ECD or domain IV (10004-H08H4, SinoBiological, Beijing, China) per well were coated on 96-well ELISA plates overnight at 4 °C. Serial dilutions of IgG or scFv were then added to the wells and incubated with the antigens for 1 h at room temperature (RT). For the competition binding assay, IgGs were mixed with serially diluted scFvs before being added to the wells containing the coated antigens. Bound IgGs were detected using horseradish peroxidase (HRP)-conjugated anti-human Fc antibodies (Invitrogen, Grand Island, CA, USA), while bound scFvs were detected using anti-FLAG-HRP antibodies (Sigma-Aldrich, St Louis, MO, USA). Following the addition of TMB solution, the reaction was read at 450 nm.

### 2.6. Yeast Display of HER2 ECD

The construction and growth of yeast displaying cells were performed as described previously with some modification [21]. Briefly, the DNA sequences of HER2 ECD and EDC variant with G427A and T450A mutations were ligated into yeast display vector and transfected into EBY 100 yeast cells. Individual yeast clones were grown, induced, and analyzed by flow cytometry.

### 2.7. Flow Cytometric Analysis

For tumor cells, a million tumor cell lines (MDA-MB-231, HCC1954 and BT474) were incubated with 1 µg of IgG Ab8 for 1 h on ice. After washing, the cells were further incubated with R-phycoerythrin-conjugated goat anti-human IgG antibody (Invitrogen) for 30 min on ice. For yeast cells, a million yeast cells were stained with 1 µg of IgG Ab8, followed by the APC-conjugated goat anti-human IgG antibody (Invitrogen) as the secondary antibody. For CAR-T cells, a million CAR-T cells were analyzed by detecting Zsgreen intensity or stained with PE-conjugated Protein L (BioLegend, Santiago, CA, USA). All cell samples were then analyzed using a BD AccuriTM C6 flow cytometer, and the data were processed using FlowJo v10 software.

### 2.8. Prediction of Anti-HER2 Antibody Recognition Epitopes

The crystal structure data of HER2 was obtained from the Protein Data Bank (PDB) (PDB ID 1N8Z). For antibody structure simulation and molecular docking, we employed Discovery Studio 2020 Software (Dassault Systems, Vélizy-Villacoublay, France) [23]. The antibody structure of Ab8 was constructed by using the “Model Antibody” module. The structure of PDB ID 5BK5 (Homo sapiens) (PDB ID 5BK5) with the highest similarity and identity was chosen as the template to construct the antibody structure. Molecular docking was conducted under rigid conditions to preserve the orientation and conformation of the molecules throughout the process. Computational resources were optimized by setting the number of parallel computational cores to 16, while all other parameters were maintained at the default values recommended by the software. The RDOCK module, a component of Discovery Studio developed for post-docking analysis, was utilized to calculate the free energy of binding of the antibody–antigen complexes.

### 2.9. Construction and Killing Assay of HER2 CAR-T Cells

The protocols utilized in this study received approval from the Human Ethics Committees at the University of Macau (BSERE17-APP020-FHS-01). The HER2-CAR construct was designed to include the CD8 leader peptide, CD8 transmembrane domain sequence, single-chain variable fragment (scFv), 4-1BB, CD28, and CD3ζ sequences. Lentiviruses carrying the CARs were generated by transfecting HEK293T cells with the backbone plasmid along with two packaging plasmids, pMD2.0G and PsPAX2. The lentiviral supernatants were collected 72 h post-transfection and filtered through a 0.45 µm filter (Millipore, Darmstadt, Germany), followed by centrifugation for 2 h at 28,000 rpm. Peripheral blood mononuclear cells (PBMCs) were isolated from buffy coats using Ficoll reagents (GE Healthcare, Chicago, IL, USA). The PBMCs were then activated using anti-human CD28/CD3 beads (Invitrogen) according to the manufacturer’s instructions. Activated T cells were transduced with the lentivirus at a multiplicity of infection (MOI) of 5 one day after activation. Spin infection was performed with polybrene at 570× *g* for 1 h at 32 °C, followed by a 2-day incubation period. On day 7, the anti-CD3/CD28 beads were magnetically removed. The activated T cells were maintained in culture medium supplemented with either IL-2 or IL-15/Rα-Fc.

CAR-T cell killing assays were conducted using the Calcein-AM release method. CAR-T cells were co-incubated with target tumor cells (BT474, HCC1954, and MCF-7 at various effector-to-target cell ratios (E/T ratios) of 20:1, 10:1, 5:1, and 1:1 in a total volume of 200 μL for 4 h. Mean fluorescence intensity (MFI) was measured using a PerkinElmer Multimode Reader at 495/515 nm.

### 2.10. Cytokine Secretion Measurement

The cytokine secretion of CAR-T cells was measured following previously established protocols [21]. The crucial cytokines (IFN-γ, IL-2, IL-4, IL-6, IL-10, and TNF-α) were determined using the BD™ Cytometric Bead Array Human Th1/Th2 Cytokine Kit II (BD Biosciences, Franklin Lakes, NJ, USA).

### 2.11. Statistical Analysis

Statistical analysis was conducted using GraphPad Prism 8 software. The significance of differences was determined using two-way ANOVA or Student’s *t*-tests. *p*-values are denoted as follows: * *p* < 0.05, ** *p* < 0.01, and *** *p* < 0.001.

## 3. Results

### 3.1. Generation of Anti-HER2 mAbs

**Figure 1:**
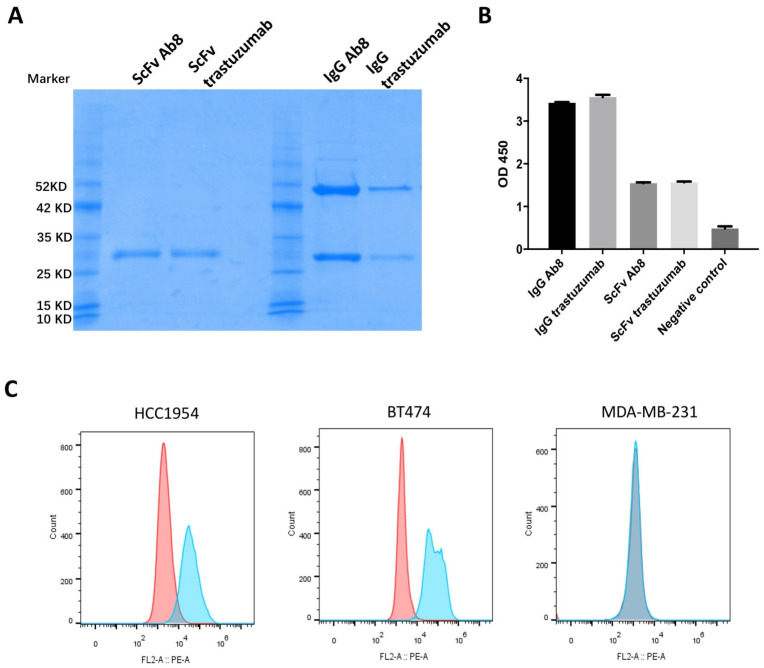
Generation of anti-HER2 mAbs. (**A**) Purity of purified IgGs and scFvs by SDS-PAGE. (**B**) Binding of IgGs and scFvs of Ab8 and trastuzumab to HER2 ECD antigen by ELISA. Antibodies were serially diluted and added to wells coated with HER2 ECD antigens. Bound IgGs and scFvs were detected with an HRP-conjugated anti-human Fc antibody and anti-FLAG-HRP antibody, respectively. The optical densities (O.D.’s) were measured at 450 nm. Data are presented as the mean ± SD (*n* = 3). (**C**) The binding of IgG Ab8 to the cell surface antigen of breast cell lines by flow cytometry. Cell lines (HCC1954, BT474, and MDA-MB-231 cells) were incubated with IgG Ab8, followed by the R-phycoerythrin-conjugated goat anti-human IgG antibody as the secondary antibody. Blue color represents IgG Ab8 staining. Red color represents control IgG staining.

To develop human mAbs against human HER2, we employed a robust approach using a large-scale naive human Fab phage-displayed library, comprising approximately 10^10^ independent clones. Recombinant HER2 ECD was immobilized onto magnetic beads and served as the antigen for the panning process. After four rounds of panning, individual phage clones were randomly selected and subjected to screening via phage ELISA against HER2. Clones demonstrating significant binding were further analyzed through sequencing. Ultimately, one clone exhibiting unique sequences was identified and designated as Ab8. The sequences of trastuzumab, a reference monoclonal antibody, were sourced from Drugbank (DrugBank number DB00072) [24]. The variable regions of heavy and light chains of Ab8 and trastuzumab are aligned (Figure S1), revealing distinct complementarity-determining regions (CDRs) of Ab8. These sequences were subsequently expressed as soluble scFvs and IgG formats (Figure 1A). Similar to trastuzumab, Ab8 is produced as a human IgG1 isotype. Both Ab8 and trastuzumab exhibited specific binding to the human HER2 antigen in ELISA assays, as depicted in Figure 1B. Flow cytometry analysis showed that IgG Ab8 recognized HER2 expressed on the cell surface of HER2+ HCC1954 and BT474 breast cancer cell lines but did not bind to the HER2- MDA-MB-231 cell line (Figure 1C). We measured the affinities between scFv formats of Ab8 and trastuzumab to HER2 ECD. Both Ab8 and trastuzumab scFvs exhibited comparable binding affinity values (9 nM vs. 10 nM) (Figure S2).

### 3.2. Different Antigen Epitope Recognition by Ab8 and Trastuzumab

**Figure 2:**
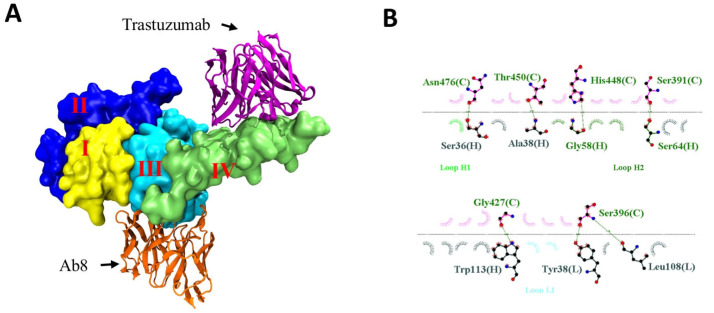
Molecular docking simulation of anti-HER2 mAbs with homology model of human HER2. (**A**) The docking model of the antibody and antigen. The four domains of Her2 are presented in different colors. The variable regions of heavy and light chains of trastuzumab and Ab8 are represented in violet and orange, respectively. The different domains of HER2 are labelled. (**B**) The predominant residues in the interaction between Ab8 and HER2.

**Figure 3:**
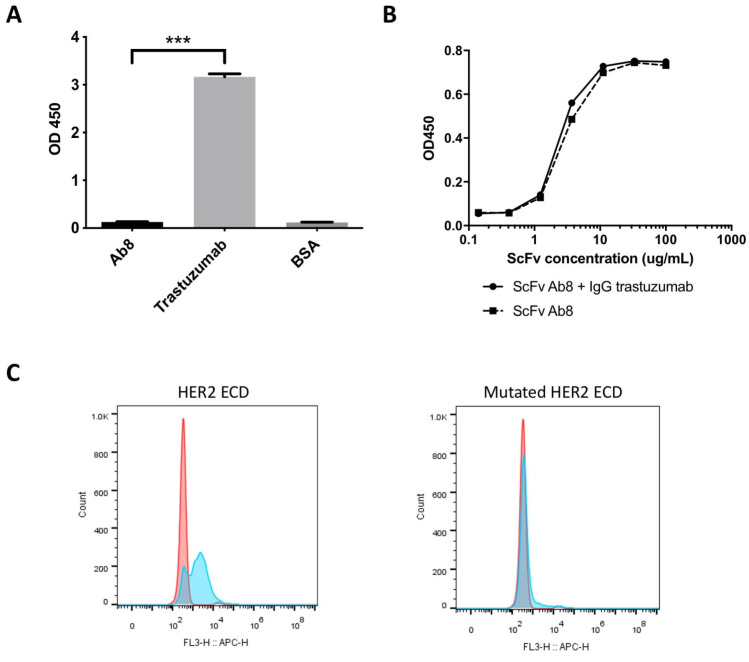
Recognition of Ab8 to HER2 domains. (**A**) Binding of IgG Ab8 and trastuzumab to HER2 domain IV by ELISA E. (**B**) Competitive ELISA of Ab8 and trastuzumab against the HER2 ECD antigen. ScFv of Ab8 were serially diluted in the presence of IgG trastuzumab and added to wells coated with HER2 ECD antigens. Bound IgGs and scFvs were detected with an HRP-conjugated anti-human Fc antibody and anti-FLAG-HRP antibody, respectively. The optical densities (O.D.’s) were measured at 450 nm. (**C**) Epitope mapping of Ab8 against HER2 on the surface of yeast cells. HER2 ECD-expressed (left) or mutated HER2-ECD-expressed (right) yeast cells were stained with IgG Ab8, followed by the APC-conjugated goat anti-human IgG antibody as the secondary antibody. Blue color represents IgG Ab8 staining. Red color represents control IgG staining. Data are presented as the mean ± SD (*n* = 3). The *p*-values were analyzed using a Student’ *t* test. (*** *p* < 0.001).

In order to elucidate the distinct antigenic epitopes recognized by Ab8 and trastuzumab on the HER2 ECD, computational methods were employed for in silico epitope prediction. Utilizing the crystal structure of human HER2 (Protein Data Bank ID 1N8Z), a homology model was generated using the Prepare Protein module in Discovery Studio. Antibody structures were constructed via the Model Antibody module in Discovery Studio. These optimized structures were then subjected to molecular docking using the RDOCK method [25]. The docking model, created with the Dock and Analyze Protein Complexes modules, was executed under rigid conditions to maintain the orientation and conformation of the molecules throughout the docking process. Computational resources were optimized by utilizing 16 parallel computational cores, while other parameters were left as default values as recommended by the software. Post-docking analysis was performed using the RDOCK module to calculate the free energy of the binding of the antibody–antigen complexes. The resulting docked model revealed that Ab8 predominantly interacts with residues 396, 391, 427, 448, 450, and 476 within domain III of the human HER2 ECD (Figure 2A,B). In contrast, trastuzumab primarily targets the membrane-proximal region of domain IV (Figure 2A), as previously reported [26]. We confirmed that trastuzumab binds to the soluble domain IV of HER2 ECD (Figure 3A). A competitive ELISA further demonstrates that Ab8 binds to a nonoverlapping epitope with trastuzumab (Figure 3B). To confirm whether Ab8 recognizes domain III of HER2, HER2 ECD and its variants with mutations at domain III G427A and T450A were separately expressed on the surfaces of yeast cells. Ab8 IgG was bound to the original HER2 ECD (Figure 3C). However, upon introducing the two mutations into domain III, the binding of Ab8 was lost. This suggests that Ab8 likely targets epitopes within domain III of the HER2 ECD.

### 3.3. Enhanced Anti-Tumor Response of CAR-T Cells through the Recognition of HER2 Juxtamembrane Domain

**Figure 4:**
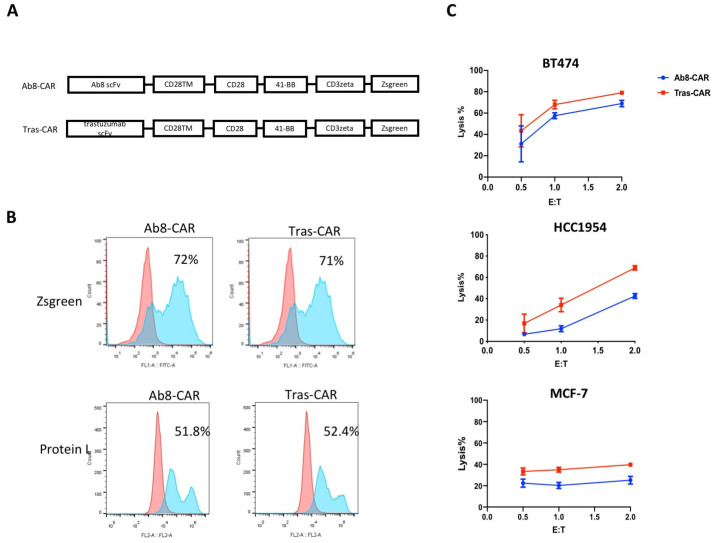
Generation of HER2 CAR-T cells and their in vitro antitumor activity. (**A**) Schematic diagram of the CAR construct. ScFv, single chian variable region; TM, transmembrane domain. (**B**) CAR expression of Ab8- and tras-CAR T cells. CAR-T cells were analyzed by detecting Zsgreen intensity or stained with PE-conjugated Protein L. Blue represents Zsgreen or Protein L staining. Red represents the negative control. (**C**) Cytotoxicity of HER2 CAR-T cells and vehicle T cells against the SK-BR-3, HCC1954, and MCF-7 cell lines. Data are presented as the mean ± SD (*n* = 3).

**Figure 5:**
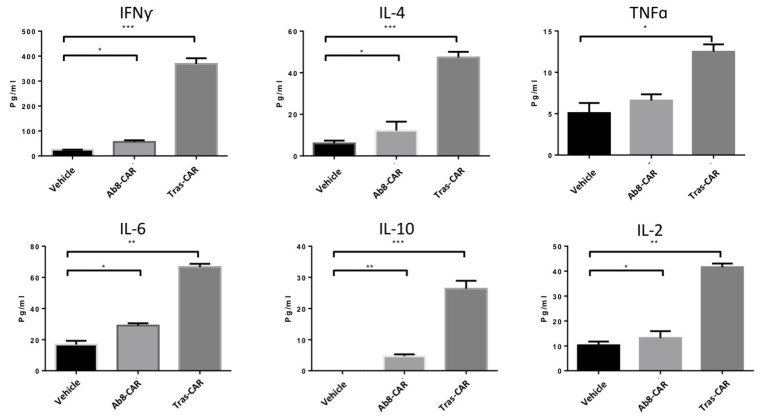
Cytokine production in HER2-CAR and vehicle T cells. Effector T cells were incubated with target tumor cells at a ratio of 2:1 for 24 h. The concentrations of cytokines were measured using a cytometric bead array. Data are presented as the mean ± SD (*n* = 3). (* *p*  <  0.05, ** *p*  <  0.01, *** *p*  <  0.001). The *p*-values were analyzed using a Student’s *t*-test.

We generated two HER2 CARs based on distinct single-chain variable fragments (scFvs) derived from Ab8 (Ab8-CAR) and trastuzumab (tras-CAR), as illustrated in Figure 4A. As shown in Figure 4B, the expression of CARs on T cells was verified by detecting the co-expressed ZsGreen. Flow cytometric analysis revealed that over 70% of T cells were positive for HER2 CARs. Protein L staining further confirmed that over 50% of CARs were expressed on the cell surface (Figure 4B). Ex vivo-expanded CAR-T cells based on Ab8 scFv were cultured with either IL-2 or IL-15/Rα-Fc, and their proliferative capacities were compared. IL-15/Rα-Fc significantly enhanced the proliferative capacity of Ab8-CAR-T cells compared to IL-2 (Figure S3). To assess in vitro cytotoxicity, the two CARs were evaluated against three breast cancer cell lines with BT474 cells (high HER2 expression), HCC1954 cells (intermediateHER2 expression), and MCF-7 cells (low HER2 expression). Tumor cells were cocultured with CAR-T cells or vehicle T cells at varying effector/target (E:T) cell ratios. As depicted in Figure 4C, tras-CAR-T cells exhibited superior antitumor lytic activity compared to Ab8-CAR-T cells. Furthermore, we evaluated the secretion of key cytokines by CAR-T cells in vitro. Following a 24 h co-incubation of HCC1954 cells and effector T cells at an E:T ratio of 2:1, tras-CAR-T cells demonstrated significantly higher levels of IFN-γ, TNF-α, IL-2, IL-6, IL-4, and IL-10 in the culture supernatants compared to Ab8-CAR-T cells (Figure 5). This finding provides the mechanistic basis underlying the differential functional responses observed between the two CAR-T cell variants.

## 4. Discussion

Recent studies have actively explored CAR-T cell therapies targeting the HER2 receptor across various cancer types, both in preclinical and clinical settings [27]. The specific recognition of tumor-associated antigens (TAAs) by antibody-bound CARs allows for the targeted elimination of tumor cells expressing these antigens. However, HER2 CAR-T cell therapies have been associated with life-threatening on-target, off-tumor toxicity [15]. To address this challenge, researchers are diligently working on strategies to improve the safety profile of these therapies. One such approach involves the careful selection of scFvs for CAR constructs. By choosing scFvs with high specificity for HER2-expressing tumor cells and minimal cross-reactivity with normal tissues, the risk of off-target toxicity can be minimized while maintaining potent antitumor activity.

In contrast to trastuzumab, which binds to HER2 domain IV, pertuzumab binds to domain II. Studies suggest that pertuzumab may be more effective than trastuzumab in preventing HER2 heterodimerization and downstream tumor signaling [5]. These findings underscore the importance of understanding the structural and functional differences between various HER2-targeting antibodies when designing CAR-T cell therapies. In our study, we sought to expand upon these insights by developing a novel anti-HER2 monoclonal antibody, Ab8, which targets domain III of the HER2 ECD.

Furthermore, to capitalize on the advancements in CAR-T cell technology, we constructed two second-generation CARs based on scFvs derived from Ab8 and trastuzumab. Significant progress has been made in enhancing the functionality of CAR-T cells to achieve improved persistence, expansion, and antitumor response. One strategy involves the incorporation of co-stimulatory domains, such as CD28 or 4-1BB, into the CAR architecture to enhance T cell activation and effector functions. Additionally, cytokine supplementation has emerged as a promising approach to augment CAR-T cell potency. IL-15, in particular, has garnered attention due to its ability to preferentially stimulate the proliferation, activation, and cytolytic activity of natural killer (NK) cells and CD8+ T cells [19]. Various IL-15/Rα complexes have been developed and studied in animal models and clinical trials [17]. The administration of IL-15/Rα has been shown to inhibit breast cancer growth by promoting cytotoxic CD8 T cells [28]. Building upon these findings, our study investigated the potential of IL-15/Rα supplementation to enhance the function of HER2 CAR-T cells. Our results demonstrate that IL-15/Rα treatment significantly enhances the proliferative capacity of both CTLL-2 cells and HER2 CAR-T cells, suggesting a potential role of IL-15 in improving CAR-T cell expansion and persistence.

When comparing the two CAR constructs, trastuzumab-derived CARs exhibited enhanced levels of key cytokines and T cell killing, highlighting the importance of scFv selection in CAR design. Despite both antibodies showing comparable affinity values, the differential epitope recognition by Ab8 and trastuzumab can lead to variable responses of CAR-T cells. Recent studies suggest that CARs targeting membrane-proximal epitopes can induce higher T cell activation compared to distal epitopes [29]. It remains consistent that trastuzumab–CAR achieves a superior response compared to Ab8-CAR. We hypothesize that the narrow binding space of trastuzumab–CAR to the proximal HER2 domain IV may exclude the phosphatase CD45 from the CAR zone, thereby enhancing T cell activation. The toxicity of “on-target off-tumor” is influenced by HER2 CAR affinities [30]. The low-affinity HER2-CAR (nanomolar level) is adequate to eliminate tumors safely and effectively, while the high-affinity HER2-CAR (picomolar level) induces lethal on-target off-tumor toxicity. Therefore, despite their nanomolar affinities, the use of Ab8 or trastuzumab may safely regress tumors.

In summary, our findings contribute to the growing body of evidence supporting the use of epitope-specific antibodies and cytokine supplementation to enhance the efficacy of CAR-T cell therapies targeting HER2-positive malignancies. Although we suggest that different epitope recognition impacts the anti-tumor response of CAR-T cells, we observed that IgG1 formats of both Ab8 and trastuzumab exhibit similar ADCC against HER2-positive cancer cell lines (Figure S4). Understanding the epitope specificity of antibodies like Ab8 and its implications for CAR-T cell responses is crucial for optimizing therapeutic outcomes in cancer treatment. Further studies are needed to fully elucidate the synergistic effects of epitope recognition and cytokine stimulation in improving CAR-T cell potency in animal models. These results highlight the potential of Ab8 as a promising candidate for inclusion in CAR constructs aimed at HER2-positive malignancies.

## Data Availability

The original contributions presented in the study are included in the article and Supplementary Materials, further inquiries can be directed to the corresponding authors.

## Supplementary Materials

The following supporting information can be downloaded at https://www.mdpi.com/article/10.3390/antib13020045/s1. Figure S1: Alignment of amino acid sequences of Ab8 and trastuzumab; Figure S2: Binding kinetics of scFv Ab8 and trastuzumab as measured with Bio-Layer interferometry; Figure S3: Bioactivities of IL-15/Rα-Fc. A. The structure of IL-15/Rα-Fc. B. The purified IL-15/Rα-Fc proteins by SDS-PAGE. IL-15/Rα-Fc consists of the long IL-15 Rα-Fc (~37 KDa) and short IL-15 (~13 KDa). C. CTLL-2 cell proliferation assay. Cell proliferation was counted by Cell Counting Kit-8 (CCK-8). D. CAR-T cell proliferation assay. Ab8-CAR-T cells were activated by anti-CD3/CD28 beads and then cultured with either IL-15/Rα-Fc or IL-2. The cell numbers were counted by the cell counter. Data are presented as the mean ± SD (*n* = 3). (* *p* < 0.05. The *p* values were analyzed using ANOVA; Figure S4: Therapy of HCC1954 xenografts with anti-HER2 mAbs.
