# Supplementary material for: Antibody Recognition of Human Epidermal Growth Factor Receptor-2 (HER2) Juxtamembrane Domain Enhances Anti-Tumor Response of Chimeric Antigen Receptor (CAR)-T Cells
Source: Antibodies (Basel). 2024 Jun 7;13(2):45. doi: 10.3390/antib13020045 (PMC11200690; doi:10.3390/antib13020045)
Supplement: Supplementary file 1 [file antibodies-13-00045-s001.zip › antibodies-2956349-supplementary.pdf]

Methods

Production of IL-15/Rα-Fc complex

Two DNA sequences encoding the sushi domain of human IL-15Rα (amino acids 1-66) fused with human IgG1 Fc and N72D mutated IL-15 were synthesized by Genewiz. Subsequently, these DNA sequences were cloned into a mammalian expression vector. The IL-15/R complex proteins were transiently expressed in 293 FreeStyle cells using PEI transfection. Finally, the complexes were purified using Protein A resins (GE Healthcare, Chicago, IL, USA).

Xenografted mouse models

The animal studies were conducted in accordance with approved protocols by the Animal Ethics Committees at the University of Macau (UMARE-018-2017). Six- to eight-week-old female NOD/SCID mice were subcutaneously inoculated with 3×10^6 HCC1954 tumor cells. Once tumor volumes reached 25-50 mm^3, mice were randomly divided into groups. To evaluate the therapeutic efficacy of anti-HER2 antibodies, IgGs were administered intravenously at four doses of 100 μg per mouse. Additionally, mice received intravenous injections of 1×10^7 human PBMCs per mouse twice. Tumor growth was monitored twice a week using digital calipers, and tumor volumes were calculated using the formula $V = ½(length \times width^2)$.

Supplementary figures

Heavy chain

```
Trastuzumab     EVQLVESGGGLVQPGGSLRLSCAASGFNIKDTYIHWVRQAPGKGLEWVARIYPTNGYTRY
Ab8             QVQLGESGGGVVRPGGSLRLSCAASGFTFSSYAMSWVRQAPGKGLEWVSAISGSGGSTYY
                :*** *****:*:**************.:..  : *************: *  :.* * *

Trastuzumab     ADSVKGRFTISADTSKNTAYLQMNSLRAEDTAVYYCSRWGGDGFYAMDYWGQGTLVTVSS
Ab8             ADSVKGRFTISRENAKNSLYLQMNSLRAEDTAVYYCARRGG--VWAFDIWGQGTMVTVSS
                *********** :.:**: *****************:* **  .:*:* *****:*****
```

Light chain

```
Trastuzumab     DIQMTQSPSSLSASVGDRVTITCRASQDVN-----TAVAWYQQKPGKAPKLLIYSASFLY
Ab8             DVVMTQSPLSLPVTPGEPASISCRSSQSLLHSNGINYLDWYLQKPGQSPQLLIYLGSSRA
                *: ***** **..: *: .:*:**:**.:      . : ** ****::*:**** .*

Trastuzumab     SGVPSRFSGSRSGTDFTLTISSLQPEDFATYYCQQHYTTPPTFGQGTKVEIKR
Ab8             SGVPDRFSGSGSGTDFTLKISRVEAEDVGVYFCMQALQTPLTFGQGTKLEIKR
                ****.***** *******.** ::.**...*:* *   ** *******:****
```

Figure S1. Alignment of amino acid sequences of Ab8 and trastuzumab.

The variable regions of heavy or light chains are aligned by CLUSTAL W software. The heavy chain CDRs are highlighted in yellow. The heavy-chain CDRs are highlighted in blue.

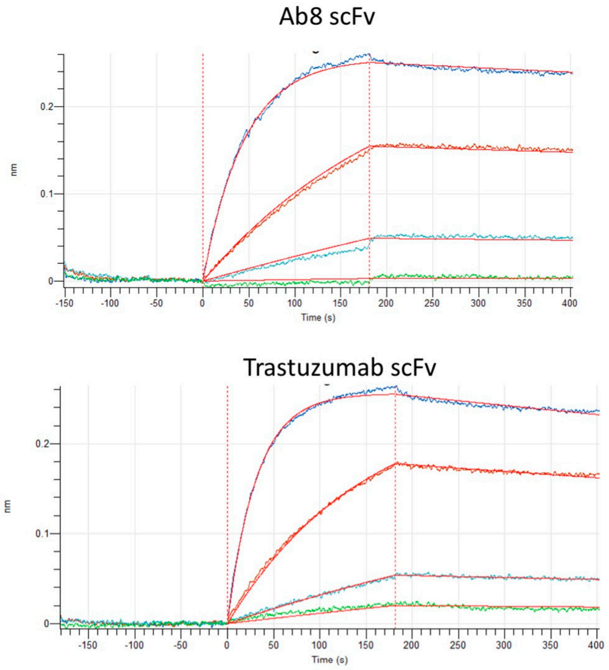

Antibody format	Ka (1/Ms)	Kd (1/s)	K_D (M)
Ab8 scFv	2.22×10^{4}	2.08×10^{-4}	9.37×10^{-9}
Trastuzumab scFv	3.14×10^{4}	4.26×10^{-4}	1.36×10^{-8}

Figure S2. Binding kinetics of scFv Ab8 and trastuzumab as measured with Bio-Layer Interferometry.

The binding affinities were performed on an Octet-K2 (Fortebio, Fremont, CA, USA) using anti-human Fc biosensors. HER2 ECD-Fc protein with PBS containing 0.02% Tween 20 (PBST) was immobilized on the biosensors at different concentrations of 1.6, 8,40, and 200 nm. Then, 5-fold serial diluted antibodies in PBST were loaded for association and dissociation. Results were analyzed by ForteBio Data Analysis v12 software.

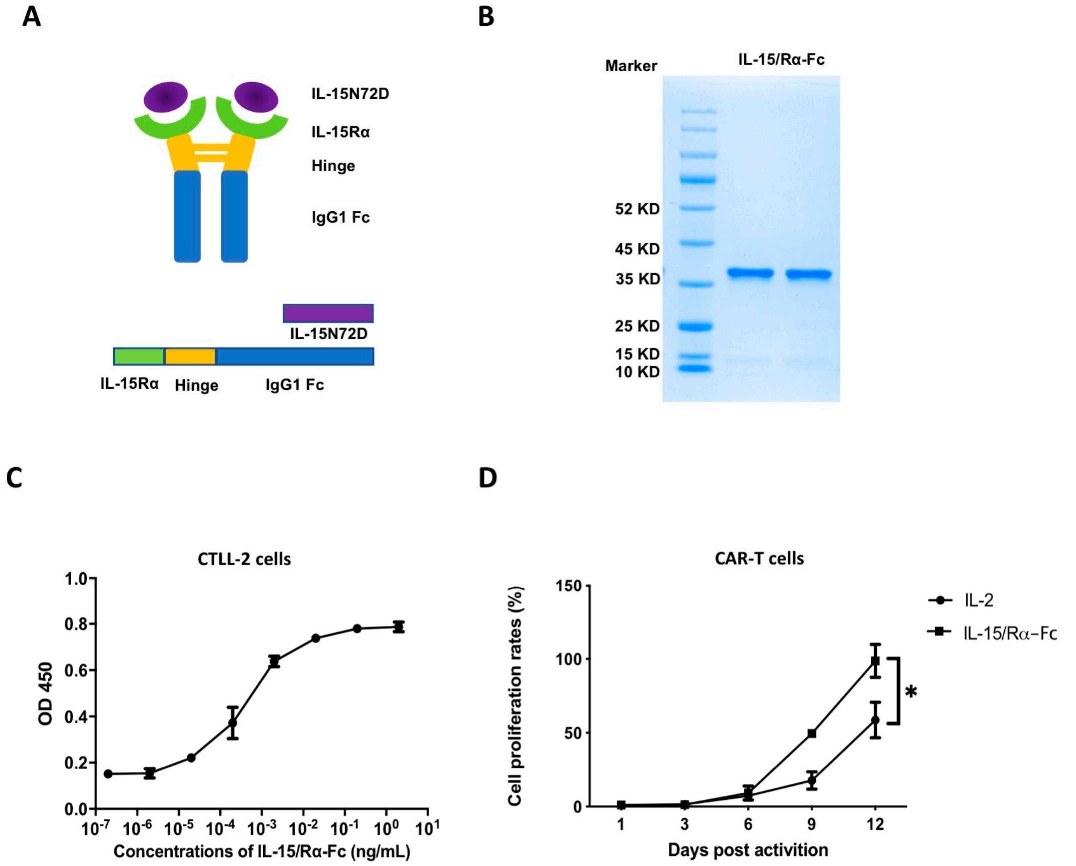

Figure S3. Bioactivities of IL-15/Rα-Fc. A. The structure of IL-15/Rα-Fc. B. The purified IL-15/Rα-Fc proteins by SDS-PAGE. IL-15/Rα-Fc consists of the long IL-15Rα-Fc (~ 37KDa) and short IL-15 (~ 13 KDa). C. CTLL-2 cell proliferation assay. Cell proliferation was counted by Cell Counting Kit-8 (CCK-8). D. CAR-T cell proliferation assay. Ab8-CAR-T cells were activated by anti-CD3/CD28 beads and then cultured with either IL-15/Rα-Fc or IL-2. The cell numbers were counted by the cell counter. Data are presented as the mean ± SD (n=3). (*$p < 0.05$). The p values were analyzed using ANOVA.

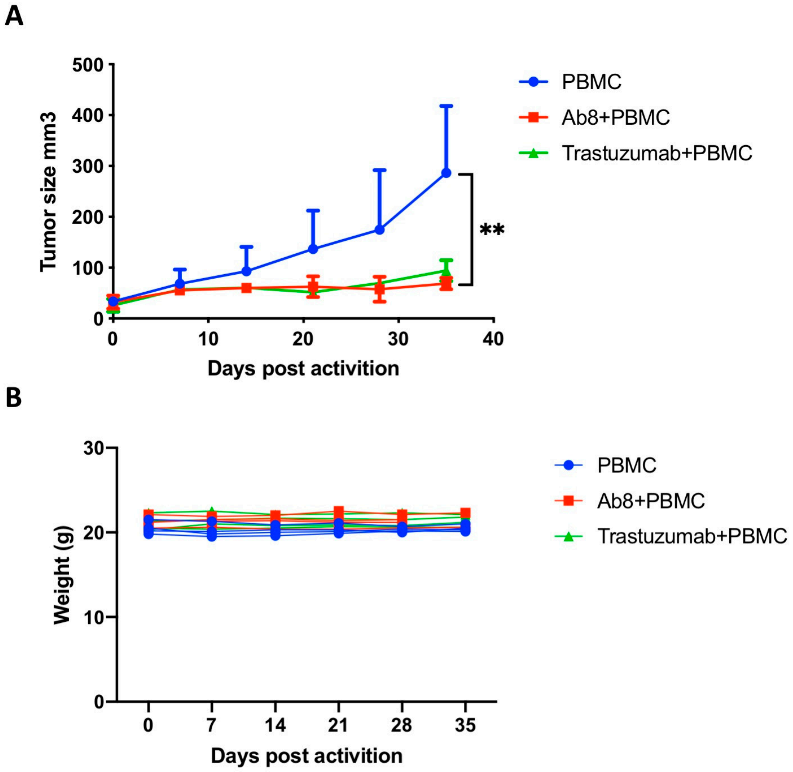

Figure S4. Therapy of HCC1954 xenografts with anti-HER2 mAbs.

Mice were inoculated s.c. with HCC1954 cells. Mice were i.v. injected with 0.1 mg IgGs (i.v., twice weekly for 2 weeks) and human PBMCs (107 cells/mice once weekly for 2 weeks). Tumor volumes (A) and weight (B) were measured weekly and are shown as the mean ± SD (n=4) (**p < 0.01). The animal studies were conducted in accordance with approved protocols by the Animal Ethics Committees at the University of Macau (UMARE-018-2017).
